# Holding Two Worlds, Keeping Art Alive: *ArtVoice* As a Method to Explore Indigenous Knowledges in *Zenadth Kes*

**DOI:** 10.1002/hpja.70245

**Published:** 2026-09-01

**Authors:** Francis Nona, Britta Wigginton

**Affiliations:** ^1^ School of Medicine Queensland University of Technology Brisbane Australia; ^2^ School of Public Health, Faculty of Health, Medicine and Behavioural Science University of Queensland Brisbane Australia

**Keywords:** climate change, decolonising research, indigenous knowledges, indigenous research methodologies, qualitative

## Abstract

Climate change is already impacting the livelihoods and lifeways of Indigenous peoples globally, including in the *Zenadth Kes* (Torres Strait) communities of the continent now called Australia. To date, Indigenous perspectives and Knowledges remain underexplored in public health scholarship. Few culturally grounded, place‐based methods exist to engage respectfully with these sensitive issues. In this article, we present the findings of *ArtVoice*, an arts‐based, community‐driven qualitative method rooted in Indigenous epistemologies and relational ways of knowing. *ArtVoice*, as a ‘method‐in‐place’, invited community members to express their experiences, hopes and concerns about climate change through creative artworks. Field visits and yarns were guided by cultural protocols, Indigenous self‐determination and cultural authority supported by a participatory approach. We present the reflections on the process and practice of applying *ArtVoice* in decolonising and culturally responsive ways, while upholding local protocols for managing and re‐representing Knowledges. This article attempts to decolonise health promotion research while simultaneously honouring Indigenous Knowledge systems. Elucidating the tensions and opportunities of these distinct knowledge systems helps us document the complexities of decolonising research practice and dissemination.

## 1 Situating Knowledges

I (FN) begin by acknowledging that all my knowledge has been handed down to me through my ancestors and for me to respectfully uphold what I have inherited, I first need to acknowledge who I am and where I come from [1]. I am an Indigenous man from *Zenadth Kes*, traditionally initiated into the customs and cultural ways of knowing, being and doing of my people. I come from *Saibai* Island with strong ancestral ties to *Badhu* Island. I pay my respects to my people of *Zenadth Kes*, particularly my *Ama* (mother). This respect extends to the community who continue to guide and shape my work. Today, my homelands are known in English as the Torres Strait Islands,^1^ the northernmost tip of Queensland, Australia.

This article offers an account of how I engage in a process of decolonising research [3]. I am supported by the suggestion that the purpose of health promotion, as per the Ottawa Charter, is consistent with Indigenous aspirations for self‐determination [4]. Hence, my writing begins with my cultural protocols. This is the foundation of any research I conduct in my homelands and in service to my people. I also acknowledge and seek to address the influence colonisation has had on my worldview, shaped in part through my western training as a Registered Nurse, Master of Public Health and doctoral research (described here). Throughout this article, the use of first‐person voice^2^ (‘I’) intentionally centres my perspective: as the Indigenous lead author embedded within the community and the research. My language choice is consistent with Indigenous epistemologies that emphasise relationality, accountability and the situated nature of knowledge [5, 6].

This article has been a collaborative process. By shifting voice through co‐writing, my co‐author (BW) and I aim to embody decolonising praxis, making explicit positionality and responsibility within the co‐construction of knowledge [7]. In our protocol article for the method described here, *ArtVoice* [8], we used the metaphor of a canoe and an outrigger to bring to life the layered nature of this research. The canoe, built by local resources and the hands of community, is guided by the principles of *Tagai*, a constellation of stars that underpin identity, order, cyclical changes and connection for people of the *Zenadth Kes*. The outrigger, by contrast, represents an outsider perspective, built to race ahead and attempt to predict and plan for what might lie in unknown waters. To convey our co‐authorship, we use this metaphor to describe the canoe as the community work unfolding in‐place, subject to the forces of the weather, tides and capacity of those on board who take lead with timing, direction, pace and destination. In some ways, I am this canoe, following protocols and self‐determination, not governing or attempting to define the destination. In other ways, I am the outrigger with my co‐author, needing to adhere to institutional requirements of research and funding deadlines, with pre‐determined destinations expected of me. As a non‐Indigenous scholar, my co‐author has worked alongside me to support this work's articulation for a western academic audience, not steering the canoe or the outrigger, but stabilising this dual process of both canoe‐and‐outrigger through editorial guidance, critical friendship and institutional co‐navigation.

Unexpected tides or waves, inherent in working as an Indigenous scholar in a colonial space, mean that in steering this canoe I am also simultaneously holding the many other canoes and outriggers out at sea navigating opposing (cultural) currents. Our co‐authorship reflects a dialogical, respectful and relational process. This is a process grounded in the cultural authority of the community and led by me as an Indigenous researcher. My co‐author's prior experience with working alongside Elders and community, as well as decolonising methodologies and critical reflexive qualitative research, supports her to work alongside me in the articulation of this method, to hear between the lines. Together, we hold the non‐duality that this work necessitates.

We begin this article by laying the context for this research and the importance of Indigenous worldviews in research. Then, we describe the study setting and context, making a case for a method‐in‐place approach to exploring climate change and Indigenous Knowledges.

## 2 Planetary Health, Climate Change and Indigenous Worldviews

Human‐driven global environmental changes pose unprecedented challenges and compounding vulnerabilities for Indigenous peoples across the world [9]. In Australia, Aboriginal and Torres Strait Islander peoples are and will continue to be, disproportionately affected by direct and indirect health impacts of climate change [10, 11, 12]. Given that Indigenous communities already face economic and social marginalisation and a higher burden of disease [13], it is crucial that any climate and health solutions centre self‐determination and Indigenous Knowledges [3]. The backdrop of colonialism, as structural, ideological and pervasive in everyday culture, economics and politics [14, 15, 16], is central to acknowledge here. So too, is the link between anthropogenic climate change and the ideologies, systems and practices of colonialism and its extractive industries [3].

Indigenous Knowledges and voices have previously been overlooked in global discourse around climate change [3, 16, 17]. This absence is evident in global reports such as the UN's Intergovernmental Panel on Climate Change [18, 19, 20, 21, 22] and the Sustainable Development Goals [23]. Part of decolonising western disciplines involves deconstructing the dominant knowledges, epistemological framing and theory that set the scene for what counts of knowledge [3, 15]. For example, the release of major reports documenting how human health depends on the state of natural systems has foregrounded the term *planetary health* [24, 25], a new term for many health disciplines [26]. In the context of health promotion, a ‘social determinants of health’ approach has been embraced at the expense of considering these ecological determinants of health [27]. Both ecological and planetary perspectives are central to Indigenous worldviews that centre holism and interconnectedness yet are slow to be taken up in dominant knowledge systems [3, 15, 16, 28]. For example, Indigenous Knowledges as a key term has been recently taken up in global reports such as the Intergovernment Panel for Climate Change [9] and the Intergovernmental Science‐Policy Platform on Biodiversity and Ecosystem Services [29].

An explicitly Indigenous perspective of planetary determinants of health was documented through a consensus process, in which Indigenous scholars, Elders, practitioners and Knowledge Holders articulated 10 determinants situated across three levels: Mother‐Earth; Interconnecting; and Indigenous Peoples [28]. In the context of climate change, it matters not just that climate impacts are documented for Indigenous peoples but also *whose* worldviews are being centred in the process. Indeed, the term ‘epistemic injustice’ offers some clarity in this context as it has been applied to climate adaptation policies to explore how they are epistemically unjust towards Indigenous peoples because of a lack of representation of Indigenous Knowledges [30]. In Australia, the absence of Indigenous Knowledges is evident when it comes to national reporting on Indigenous health and wellbeing. For instance, the recent Health Performance Framework Summary Report acknowledges cultural determinants of health including ‘caring for Country’ and ‘access to traditional homelands’, yet in reporting these determinants remain absent while ‘social determinants’ (housing, education, income, employment and socioeconomic indexes) of health are reported on in detail [13].

It has been acknowledged that Indigenous Knowledges cannot simply be ‘merged’ with western scientific knowledges [28]. The ontological (ways of being) and epistemological (ways of knowing) roots of these distinct knowledge systems mean their integration or combined application is complex. This was termed the ‘Cultural Interface’ and described a ‘multi‐layered and multi‐dimensional space of dynamic relations constituted by the intersections of time, place, distance, different systems of thought, competing and contesting discourses within and between different knowledge traditions and different systems of social, economic and political organisation’ [31] (p. 199). Our research is situated at the Cultural Interface, where I attempt to translate Indigenous Knowledges into a western format.^3^ I do so to show the merit of holding both ways of knowing alongside one another, in their multi‐layered and multi‐dimensional complexity of relations, neither complete nor superior [31].

Indigenous Knowledges in this context are explored through a newly developed arts‐based qualitative method called *ArtVoice* [8]. Informed by scholars advocating for ‘methods‐in‐place’ [32] and art as truth‐telling [33], I conceived of *ArtVoice* to explore community members' experiences, hopes and concerns about climate change expressed through art. It was during my field visits that I learned of the challenge that sat before me: how do I represent communities' Knowledges and voices ‘out‐of‐place’: that is, in written form, de‐contextualised from the lands and peoples it is embedded. Hence, this article aims to document the process of representing Indigenous Knowledges and upholding cultural protocols that seek to protect and honour community experiences, hopes and concerns. In this way, this paper holds both canoe and outrigger and offers an example of decolonising climate change Knowledges at the Cultural Interface.

## 3 Research Setting and Context

This research was carried out in *Zenadth Kes*, which is located at the top end of Queensland, Australia. The region is made up of an area of around 48 000 km^2^ of open sea, with over 90% being ocean [34]. SeaCountry plays a critical role in Islander cultures, as it provides hunting and fishing resources for a subsistence‐based livelihood and is also an integral part of Islander identity, contributing to our spiritual and physical well‐being [35, 36, 37, 38, 39].

Despite arguably contributing least to global emissions, communities across *Zenadth Kes* continue to experience disproportionate climate impacts and have sought justice through landmark legal actions at national and international levels ([40, 41]). These historic Torres Strait‐led cases are part of a broader movement advancing self‐determination, legislative reform and environmental justice as one upstream public health response holding governments to account [42]. My participatory and community‐based doctoral research exploring Indigenous Knowledges is situated within these ongoing assertions of cultural sovereignty, self‐determination and protection of homelands in the face of climate change. A participatory approach that prioritises community to lead with cultural protocols is consistent with Indigenous protocols of self‐determination and sovereignty [43, 44].

## 4 Methods

Ethics approval was obtained through the university (#UQ2022/HE002188). The work is governed by the NHMRC's Ethical Conduct in research with Aboriginal and Torres Strait Islander Peoples and Communities [45] and the AIATSIS Code of Ethics for Aboriginal and Torres Strait Islander Research [46]. The protocol and method development for *ArtVoice* has been previously published [8]. *ArtVoice* is an arts‐based, community‐driven qualitative method rooted in Indigenous epistemologies that invites community members to express their experiences, hopes and concerns through creative artworks [8].

Art and storytelling are longstanding cultural practices across the islands, supported by regional art centres. Artists from the islands engage with a wide variety of creative mediums, including printmaking, etching, textiles, jewellery and carving. The chosen art centre, an established Indigenous corporation with longstanding regional connections, provided a relational and culturally grounded setting for the study. Initial engagement was undertaken with an island‐based art centre to seek permission to conduct the research within the community. Participation was not limited to established artists; it was open to all community members interested in being part of an art‐based project exploring Indigenous Knowledges and climate change. Verbal consent will be sought from each participant, followed by one written consent (signed participant and consent form). The emphasis is on verbal consent given the strong use of storytelling processes consistent with Indigenous Research Methodologies. Two field visits have been conducted to date. Both trips prioritised cultural protocols, deep listening and yarning to facilitate the co‐construction of meaning. Engagements were guided by respectful, reciprocal and responsible decolonising practices [6, 47].

The first field visit to *Badhu* Island took place in September 2025, with the purpose of seeking a ‘blessing’ from Elders and community leaders for the research. This involved me undertaking a listening process with key community leaders to shape the scope of the research and refine *ArtVoice* in collaboration with the community. It was in this initial visit that I approached potential participants to explore their interest in being involved in the project and what it might look like for them to be involved. These initial yarns were fundamental to establishing a relational stance in the research, giving community voice and agency in the research process and ultimately the outcomes [48, 49].

The second field visit took place in February 2026. The second visit involved both myself and my co‐author with the aim of visiting to listen and learn from community about their stories and art‐making. Relationships were central to this visit and had their own pace and tempo that I needed to respect. We were embedded in community day‐to‐day life on the island. Through my two‐week visit, I witnessed my relationships deepen and my perspectives of my research and role shift and take shape. The notion of ‘data collection’ as a discrete and formalised ‘moment’ that is documented or captured in some way was something I navigated and held throughout my visits and is discussed in more detail in the findings to follow. My situatedness as both community and researcher meant I was navigating a fine line between the canoe and the outrigger and yet yarns about art, Knowledges and experiences of climate change were daily.

## 5 Analysis

Decolonising the research process requires careful reflection on what ‘analysis’ means. *ArtVoice* has been a process of reallocating power to Indigenous peoples and Indigenous ways of knowing. This method allows me to honour the living and inherited Knowledges that have sustained my people for thousands of years and that deserve to be heard and translated (in line with protocols) to western audiences. I conceived of *ArtVoice* within an Indigenous Research Methodology as a ‘vessel’ of translation, one that sits outside of the colonial frame and tools, that supports expression across time and space and that honours the oral and artistic traditions to which I am embedded.

In describing my process of ‘analysis’, I join Indigenous writers attempting to language our ways within western formats, or as *St'at'imc* and *Celtic* scholar Cole (2002) puts it, ‘take[ing] up the tools of the settlers’ despite the fact that the ‘idea of a paragraph is meaningless to my sense of oral contiguousness with the land with community with acting in the world’ (p. 448). Similarly, the notion of extracting units of meaning from a bigger story that is situated within a relationship is foreign to me. Pacific‐Indigenous researchers have critiqued widely applied qualitative methods, such as thematic analysis, as linear and requiring an external observation of tangible, categorised data which sits at odds with their cultural ways of knowing as lived, intergenerational, non‐linear, holistic and spiritual which all become interwoven into the analysis [50]. Those authors draw on the metaphor of weaving to highlight the difference between the creative and ethereal processes of Indigenous ways of knowing and western ways of knowing, the latter were likened to a ‘brick‐build house’ [50]. Living metaphors are often deployed in explaining Indigenous research methodologies to western audiences [48].

As an Indigenous researcher, analysis for me, then, is an inherently decolonial process. Analysis is an extension of my Indigenous protocols and ways of gathering and disseminating knowledges [51]. To be clear, it is not a discrete phase that requires expert/cognitive labour or external procedures of pre‐defined rigour. Rather, it is an ongoing, emergent, embodied and flexible process of coming to understand the topic [52]. Analysis is embedded within my ways of knowing, being and doing in which my relational accountability and lived connections to my people is central [53]. Data too are not some external or objective ‘thing’ that can be removed, concretised and coded following external theory or tools that are deemed ‘worthy’. Analysis is inter‐subjective, embedded within a web of relationships, born out of worldview and lived experience [50]. Hence, analysis was an ongoing, dialogical and relational process. Indigenous peoples have always conducted research, yet writing about it is relatively new within our historically oral societies [51]. Such historical differences in knowledge generation and dissemination create an inequity today. For instance, I cannot cite my ancestors or Elders using a western‐style referencing system, for they have not published in peer‐reviewed academic journals. The style of ‘writing up’ the analysis reflects the relational, non‐linear ways of knowing that we are trying to honour.

Contrary to dominant understandings of research, in this project there is no distinct classification between data collection and analysis. Rather, these were simultaneous and overlapping. My ‘analysis’ unfolded through the two field trips, yarning with Elders and community, sharing, listening and reflecting and being on Country. All of these practices reflect a way of being that as an Indigenous researcher requires me to be steeped in the place, with my people [48] and only through this process of immersion allows me to offer the ‘findings’ presented here. Similarly, dualisms between a methods paper and an empirical findings paper are blurred here in this article. Steeped in the personal and cultural worlds of my people, the ‘findings’ reflected here may read to the western audience as ‘autoethnography’, for this may be the method that comes closest to the relational genre my work embodies.

Another layer of what supported this articulation of ‘findings’ was sitting with my co‐author before, during and after field visits. It was during these largely unstructured yarns where I was able to reflect and dialogue in relationship and at the Cultural Interface, as I attempted to translate (across languages, worldviews and epistemological frames) my account of what I was observing through this research. What is represented in this article is a synthesis of my field visits and yarns on‐Country and the protocols I am tethered to as an Indigenous man. I attempt to speak to the ontological divide between Indigenous and western knowledges and in doing so I present the ‘findings’ of *ArtVoice* in a tone that is deliberately reflective, arising from an embodied process. To honour the journey and support our readers to witness the insights of the canoe (those that emerged in‐place, in‐relationship and through yarning), we have chosen to structure the findings around these two field trips.

## 6 Findings

### 6.1 Field Visit 1—‘Blessing’: Decolonising by Returning to Relation

On Trip 1, I confronted the coloniser's gaze in the impulse to ‘extract’ art or even conversation. I saw in this a tendency to abstract such moments as *it*, as ‘object’ and hence position ‘it’ as data. These data, I came to see, were the ‘destination’ of the research; it seemed to be about getting to this pre‐defined place, where the ‘gold’ lay. This was evident in the questions I received from university colleagues over the course of this research: ‘what is your data?’ I could not quite locate why this question made me feel uncomfortable and yet it took me being in situ with my people to see the gaze I had learned through the institution to understand the discomfort.

Through yarns and time sitting in cultural protocols, I began to learn that removing art from relationship, archiving it outside of kin, Country and protocol deadens the art. This trip reoriented me to the truth that art lives through connection with our people and culture; interpretation cannot be outside the artist. My role is to facilitate interpretation with artists and community, not to impose a solitary ‘so what’ or pre‐defined destination. In short, this trip supported me to step into relationship and learn to see how extractive I could become if I did not prioritise cultural protocols. I was reminded that Indigenous Knowledge is not an abstract object to be ‘known’; it is felt. The work, then, is not to strip knowledge of its relational life through abstraction. Trip 1 also sketched a program of work for me; I needed to lead with cultural knowledges first and to use *ArtVoice* as a method that speaks with (relationship‐to), not ‘about’. In short, this trip re‐grounded the research in relation, feeling and protocol and shifted my stance from collector to facilitator and custodian of Knowledges.

#### 6.1.1 Parallel Processes and Accountabilities

**FIGURE 1:**
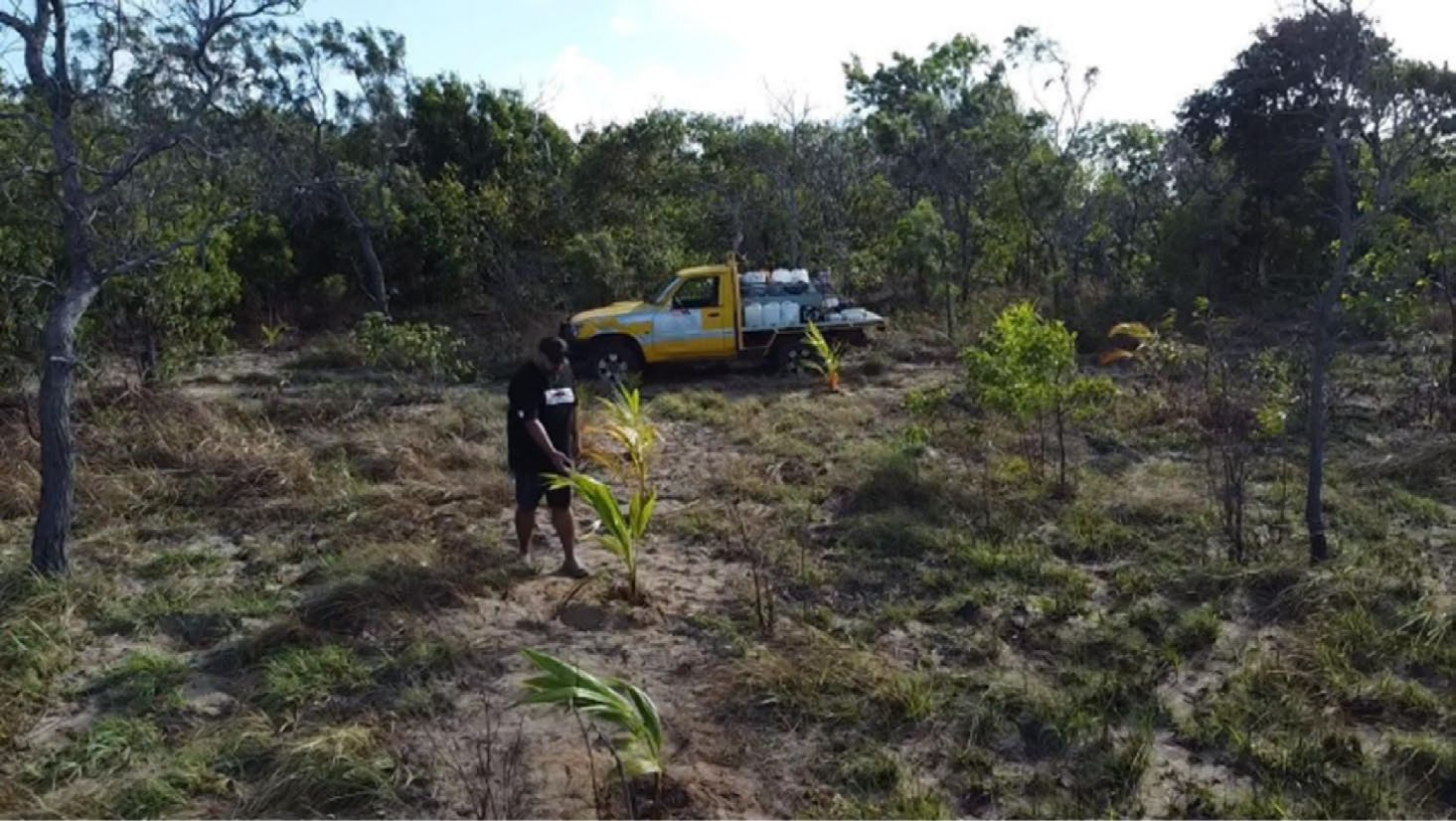
Trip 1: FN involved in cultural practices. Photograph taken September 2025.

I recognised a parallel process unfolding for me in this trip, that is: the Western PhD alongside a PhD in my culture. The latter included a deeper, personal call to be a relational bridge for my son. As part of my cultural protocols, I engaged in cultural practices that involved planting young coconut trees on‐Country (see Figure 1). This process, a long‐standing cultural practice, then emerged as the metaphor of that bridge that my presence and being on the island was creating. This further reframed the outputs that were not only held in text or data, but living through protocol and kinship. My visit on the island, I realised, was not just for my PhD research; it was part of a bigger journey of bridging my son back to our homelands. While seemingly peripheral to the project, this further deepened my relationship with and commitment to, Country and my people and inevitably shaped my project moving forward.

### 6.2 Field Visit 2—‘The Mirror’: Protocol First and the Pace of Relation

#### 6.2.1 Wayfinding Outside the Project Frame

Trip 2 unfolded at the ‘speed of trust’. By this, I mean that I spent days yarning with a world‐renowned artist, building rapport and finding a waypoint outside the project frame to meet each other properly. This slowed ‘data collection’ while accelerating relationship. Part of this time with the artist centred around a pivotal decision to *not* record our yarns. That choice looked detrimental to a conventional research project, yet it invested in relationship and allowed me to arrive as a community member, not merely a researcher there to ‘conduct the research’ and ‘extract the data’. The six‐hour sitting (with only an hour recorded on an audio device) laid foundations that could not have been built through an extractive posture. It was in relationships such as these that I began to reflect on the two PhDs unfolding in parallel, each mirroring the other. In my reflective notes I wrote:

I am holding two parallel PhDs:

Community say: ‘you have the PhD already. I will give you strength, if you find it within.’

Academia says: ‘the PhD is external.’ It is a process of looking for validation externally to assert a strength, a knowing. They ask: ‘where is the data’ as though data is something separate from me. (FN's Reflective Notes, Feb 2026)

I became aware that the Western world is a changing climate that often extracts and drains. I have found my own experience as an Indigenous scholar a draining and extracting experience. I lean on Indigenous scholars' articulations [48] to ground me here, reminding me that I am not imagining the weight of this workload I carry, these parallel PhDs. Each return home makes me strong because reciprocity is embedded there. That said, this second trip clarified that serving my people is the only way I stay strong as a researcher and that the PhD must ultimately be in service to community, not the other way around. Trip 2 translated the lesson of relation into practice and following my cultural protocol set the pace and tempo. I learned that recording ‘data’ is secondary to right relationship and that worthiness is grounded within, not conferred by institutional validation.

### 6.3 In Reflection: Managing Knowledges, Balancing Both‐Worlds

Across both trips back to the island, one key understanding holds: art lives only in relationship. Outside of relationship, ownership and abstract interpretation deaden it. This was told to me in many ways over the course of my visits, as I sat with artists yarning about their works. I learned that meaning is brought to life with the artist and community through a facilitated interpretation (or translation). This single learning has profound implications for my research. Most obviously is that I risk being complicit in deadening my peoples' art by owning, abstracting or de‐contextualising it through written research in English that will be held and stored on the electronic shelves of western institutions.

In my research, the ethical task is not to ‘share’ knowledge but to manage it with care, in line with my protocols and responsibilities. Sitting at the Cultural Interface means I must recognise that institutions often draw from Indigenous people in ways that generate benefits for the academy while creating burdens or costs elsewhere and often unseen [14]. Within this context, terms like *Indigenous Cultural and Intellectual Property (ICIP)* can be taken up superficially or used to reinforce institutional control, treating knowledge as something that can be owned, displayed, or traded. Protocol teaches me otherwise: some things are not to be spoken, not because they lack value, but because their value is held in the integrity of relationship. Knowledge that is carried and led by us does not depreciate; it remains alive through connection, not circulation. Managing knowledge, then, becomes an act of sovereignty: deciding not only *what* enters the research, but where Indigenous knowledges are planted, tended and kept alive.

Following the earlier imagery and cultural practice of planting coconut trees, these reflections on managing knowledge raise the question: ‘Where do I plant the coconut tree?’ This captures the choice before me: plant my peoples' knowledges in the university's foyer where it can be displayed and tended by institutional gardeners, or plant on the riverbank of Country where my kin can water it. I have learned that one can keep the tree alive, the other deadens it. This is not just a metaphor. This question aligns with questions posed by *Kahnawà:ke Mohawk* activist and scholar Alfred [14]: ‘The question for Indigenous academics is this: are we part of the process of destruction of Indigenous cultures and nations, or are we upholding our responsibility to contend with it? What can we do? And what is the way to transcend this situation and regenerate our communities and cultures so that our peoples may survive into the future? (p. 93)’.

## 7 Discussion

The purpose of this article was to document the process of representing Indigenous Knowledges and upholding cultural protocols that seek to protect and honour community experiences, hopes and concerns. In doing so, we have attempted to hold both canoe and outrigger and offers an example of decolonising climate change Knowledges at the Cultural Interface [31] in applying *ArtVoice. ArtVoice* is a qualitative arts‐based method that is Indigenous‐led, embedded in Country and accountable to Elders who guide protocol across design, data‐generation, analysis and dissemination [8]. This article documented my meaning‐making process that unfolded through yarns, being on‐Country and in relationship with cultural protocols. Meaning‐making was culturally situated and simultaneously deconstructed at the Cultural Interface, rather than imposed from outside or dictated by western ‘best practice’ guidelines or expectations. This *method‐in‐place* [32] and the reflections documented here were informed by decolonising principles, including relationality, reciprocity and responsibility [7, 48]. In this way, this article blurs dualisms and presents both methodological and analytical insights of working on‐Country and at the Cultural Interface.

I can see now that these two field visits re‐calibrated both my research and my role as an embedded researcher who is both of the community and the academy. Trip 1 served to decolonise my research and by this I mean that it turned me back towards cultural protocols as first and foremost. I found that I returned home recalibrated, mirrored by community as a leader in my own right and was reminded that the living potential and aliveness of our art is held in relationship, not in files, museum galleries or manuscripts. By the time I returned, Trip 2 deepened this gradual shift in my perspective and way of seeing my research by slowing me down to the tempo of protocol. By this I mean the long and non‐linear yarns, careful wayfinding and a deliberate choice to prioritise relationship even when it appears ‘non‐productive’ to the research. Together the trips taught me that my task is not to extract or own the knowledge of my people, but to remain in relation with it, managing knowledge responsibly, allowing it to be a vessel of truth‐telling [33]. My field trips raised the ultimate and most challenging, question for me to hold while doing a PhD within a western context: ‘what is an ‘output’ when the researcher is the work, embodying rather than producing abstracted knowledge’?

*ArtVoice* has been my attempt to find a middle ground between Indigenous and western knowledge systems, where they can meet on equal footing. Nakata's [31] theoretical concept *the Cultural Interface* offers language of a transformative dynamic between people and/or institutions from differing knowledge paradigms (i.e., Indigenous and western) seeking to work together. What we have learned through this process is about relationship. The canoe and the outrigger are interdependent. When one crashes, so does the other and hence we need both to work together. Similarly, our co‐authorship necessitates a working together, an unpacking of all views and possibilities, a ‘hearing each of us out’ and sitting through any discomfort or vulnerability that arises from this. The purpose of this unpacking and deconstructing is to ensure any unconscious harm or misrepresentation is tended to. In my words, ‘if you don't hear it, see it, then right there it becomes dangerous’. Together, we tried to see, hear and feel into what may not be seen or known. This is not some destination to arrive at; it is a process that brings our humanity, vulnerability and interconnectedness to the fore. We see that health promotion research may benefit from foregrounding and embodying such processes, being open to unknowing and unlearning and the transformative potential when a multiplicity of knowledges are held together.

Attempting to hold both worldviews and find a balance has led me to reflect on questions posed by Alfred [14] about my role as an Indigenous academic. The battlefield of universities as sites of personal gain and individual recognition [14] sit at odds with who I am as an Indigenous man and why I joined academia. Hence, I see my role as an embedded researcher held between two worldviews as one of *knowledge stewardship*, where decisions about what can be shown or told are led by community and governed by Indigenous ethics. For my research, this meant that artworks and stories remain living in right relationship rather than being extracted as institutional property. I write in first person to make my positionality answerable to both community and academy, translating felt knowledge into forms legible to Western readers without compromising Indigenous authority, sovereignty, or protocol. This unique style of writing and reporting on research enables me to walk between two worlds with integrity and respect. The shifting of voice from ‘I’ to ‘we’ offers perspective of how we hold the cultural interface and the complexities named in this article through co‐writing.

We hope that these findings may inspire future arts‐based, place‐specific work that is rooted in local cultures and protocols. *ArtVoice* is not a method that can be lifted out and applied to any peoples, culture or context; the cultural authority structures, kinship protocols and relationship to Country that shape this work are specific to *Zenadth Kes* and cannot be transferred. What may transfer is not the method's content, but our stance: being led by community protocol rather than researcher agenda; and prioritising relationship and process over the extraction of ‘findings’. It also resists the pull towards proceduralism, what Chamberlain [54] terms ‘methodolatry’, that many western qualitative methods privilege. In this sense, *ArtVoice* becomes a ‘container’: shaped by and answerable to, the protocols, traditions and people of this place. The method itself becomes the vessel for decolonising research, for holding accountability to place and people, rather than a set of steps for others to repeat.

As a final point, I note that the work presented here prioritises *process* over content. This is because, within an Indigenous frame, knowledge is lived, felt and relational, not extracted or owned [55]. Presenting the findings in this way is informed by the recognition of the colonial backdrop and that Indigenous Knowledges must be *managed* carefully rather than shared indiscriminately. Some things can be spoken, some cannot and the value of protocol remains constant even when academic pressures and processes encourage disclosure. My findings therefore do not present themselves as abstracted data points, but as a record of relational insights, cultural responsibilities and the ongoing negotiation required to keep Indigenous Knowledges alive within western research settings. This article is an attempt to foreground the Cultural Interface as part of attempting to redress the epistemic injustice surrounding Indigenous Knowledges in the context of climate adaptation [30]. Foregrounding the journey of the canoe, this article is intended to stir reflections from western, non‐Indigenous researchers seeking to learn about an application of the Cultural Interface as part of decolonising health promotion [4]. We invite researchers to observe their ways of relating to Indigenous Knowledges as a first step to decolonising their practice. This may include noticing the pull towards familiar, legible forms of knowledge and choosing instead to stay in the discomfort, uncertainty and un‐knowing the canoe's journey demands.

## 8 Closing

We are dancing, fighting, to a drum that is felt more than heard. (FN's words).

When I remain in relation to art, it lives; when I am asked to own or extract it, it dies. The benefits of centring this truth are evident for community and, if listened to properly, for society. *ArtVoice* is my commitment to plant and tend the coconut tree where it will live, on Country, in relation, stewarded by protocol, so that my son and our people, can keep dancing to a drum that is alive in us.

## Funding

This work was supported by the National Health and Medical Research Council (The Healthy Environments and Lives Innovation Fund 2024).

## Ethics Statement

Ethical approval has been received from the University of Queensland Human Research Ethics Committee (reference 2022/HE002188) as part of a wider project examining the health impacts on Aboriginal and Torres Strait Islander Peoples from climate change. The work is governed by the NHMRC's Ethical Conduct in research with Aboriginal and Torres Strait Islander Peoples and Communities [45] and the AIATSIS Code of Ethics for Aboriginal and Torres Strait Islander Research [46].

## Conflicts of Interest

The authors declare no conflicts of interest.

## Data Availability

In line with Indigenous Cultural and Intellectual Property, all the art and Cultural Knowledges produced through this research are owned by the community (and community members) and not the researcher or institution. Any sharing or dissemination of art produced by participants is ultimately the decision of participants, Elders and the community who ultimately own the research and knowledges. Given the complexities of Indigenous data and knowledge and nuances around this knowledge sharing process, no data will be made publicly available.

## Endnotes

^1^
The Torres Strait Islands is named after Captain Luis Baes de Torres, the Spanish mariner who “discovered” Torres Strait in 1606 [2]. In this article, we use *Zenadth Kes* (as a traditional place name) and Torres Strait Islands interchangeably, including to refer to the peoples. We acknowledge the need for historically situating language use, especially within a research project intending to decolonising language and Knowledges.

^2^
We deliberately use of first‐person language as this is consistent with Indigenous research epistemologies that prioritise relationality, accountability, and positioning [5]. This is a co‐authored article, so we have chosen to centre the first author's (F.N.) voice, standpoint, and authority where it is culturally important to do so. The second author (B.W.), who is a non‐Indigenous researcher, has contributed through collaborative writing, editing and academic guidance, with deep respect for the cultural foundations of this work. While the research is embedded in Indigenous epistemologies, this collaboration also reflects the navigation of constraints and possibilities inherent in publishing Indigenous‐led research within academic institutions where the support of a critical friend and co‐author supports an articulation across cultures.

^3^
This ‘format’ necessitates western processes of knowledge legitimation, including that it is written in an English and peer‐reviewed by outsiders to the community and context.
